# Supplementary material for: Chromosome compartments on the inactive X guide TAD formation independently of transcription during X-reactivation
Source: Nat Commun. 2021 Jun 9;12:3499. doi: 10.1038/s41467-021-23610-1 (PMC8190187; doi:10.1038/s41467-021-23610-1)
Supplement: Supplementary file 1 — Supplementary Information [file 41467_2021_23610_MOESM1_ESM.pdf]

Supplementary Information

# Chromosome compartments on the inactive X guide TAD formation independently of transcription during X-reactivation

Bauer *et al.* 2021 Nature Communications

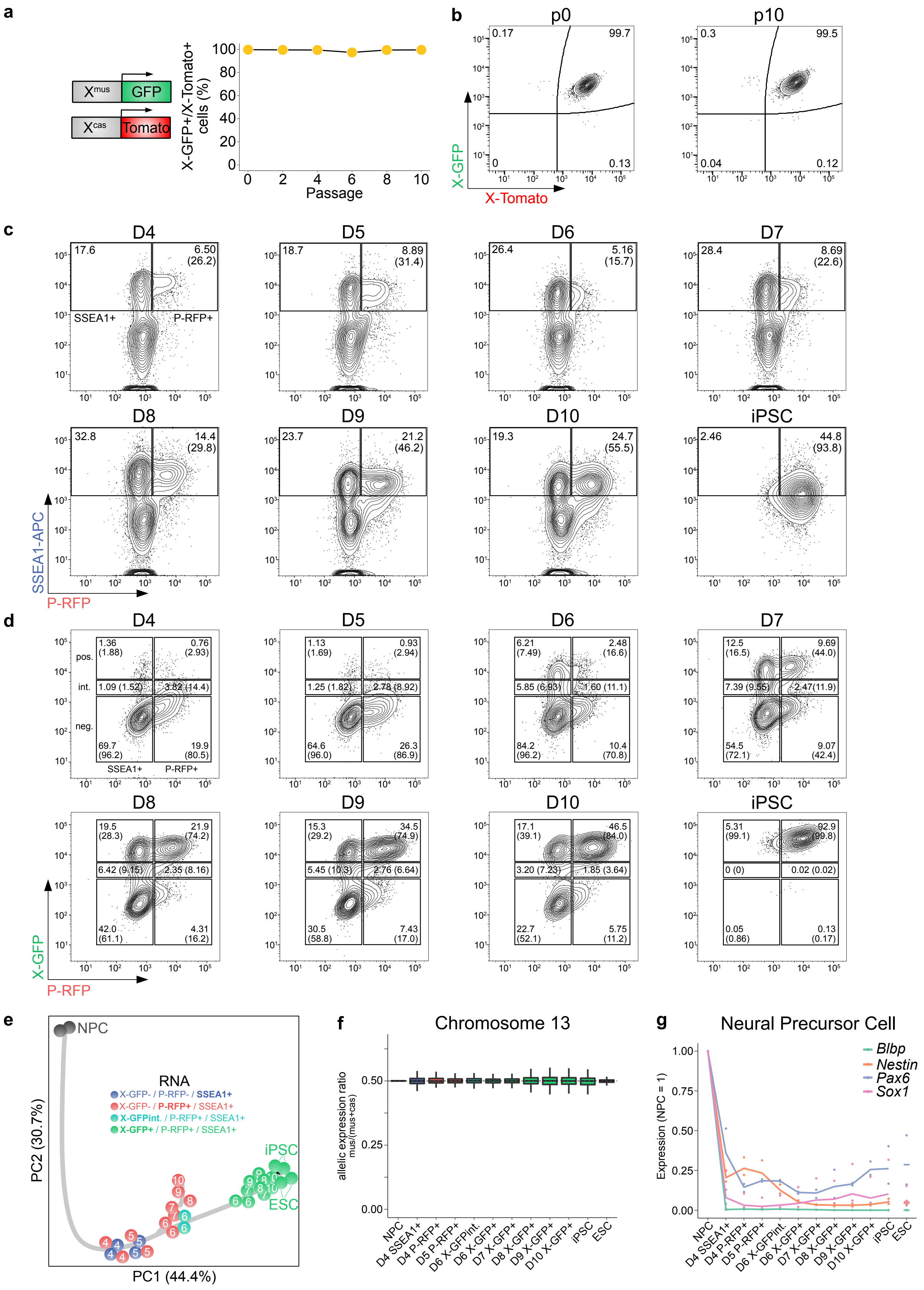


**Supplementary Fig. 1 A tailor-made reprogramming system to efficiently trace X-chromosome reactivation.**

(legend on next page)

**Supplementary Fig. 1 A tailor-made reprogramming system to efficiently trace X-chromosome reactivation. a** Left, schematic of the X dual color reporter. Right, quantification of X chromosome loss in X dual color ESCs cultured in serum plus LIF conditions for 10 passages, with passaging every other day. **b** FACS gating strategy of X-GFP and X-Tomato reporter expression at passage 0 and 10. Shown are representative contour plots gated on live cells. Numbers indicate the percentage of cells. **c** FACS gating strategy of SSEA1 and P-RFP reporter expression during a reprogramming time course. Shown are representative contour plots gated on live cells. Numbers indicate the percentage of cells. Numbers in brackets indicate the percentage of P-RFP+ cells out of SSEA1+ cells. **d** FACS gating strategy of P-RFP and X-GFP reporter expression during a reprogramming time course. Shown are representative contour plots gated on SSEA1+ cells in (**c**). Numbers indicate the percentage of cells. **e** PCA of dynamics of gene expression during reprogramming including neural precursor cells (NPCs) (n = 12,318 genes). Solid grey arrow, hypothetical trajectory. Dashed grey arrow, samples deviating from main reprogramming trajectory. **f** Allelic expression ratio (mus/(mus+cas)) of protein-coding genes expressed from chromosome 13 (n = 335). Biallelic expression, ratio = 0.5. Box plots depict the first and third quartiles as the lower and upper bounds of the box, with a band inside the box showing the median value and whiskers representing 1.5x the interquartile range. **g** Average gene expression kinetics of neural precursor cell markers *Blbp*, *Nestin*, *Pax6*, and *Sox1* during reprogramming (n = 2, relative to the levels in NPC).

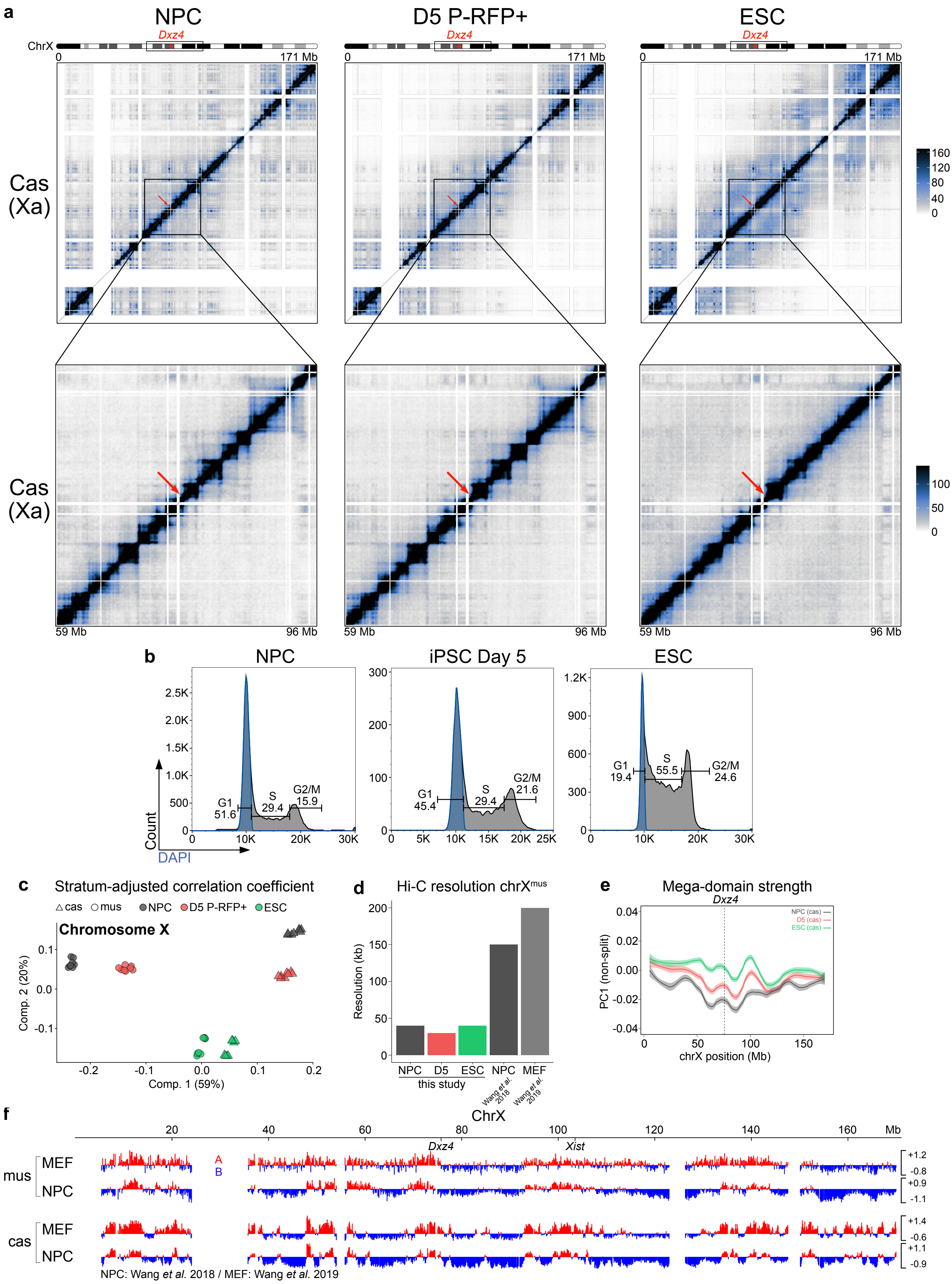


**Supplementary Fig. 2 The inactive X chromosome exhibits A/B-like compartmentalization.**
(legend on next page)

**Supplementary Fig. 2 The inactive X chromosome exhibits A/B-like compartmentalization.**
**a** Allele-specific Hi-C maps of the always active chromosome $X^{cas}$ across stages. Top: Entire chromosome is shown at 200-kb resolution. Bottom: Zoom-in of the mega-domain boundary is shown at 100-kb resolution. Scale is shown in mega-bases (Mb). The mega-domain boundary *Dxz4* is indicated by a red arrow. White-shaded areas, unmappable regions. **b** FACS analysis of cell cycle using DAPI. Blue shading indicates sorted G1 population. Numbers indicate the percentage of cells. **c** Correlation of Hi-C samples at 100-kb resolution using stratum-adjusted correlation coefficient[1], visualized as a multi-dimensional scaling plot. **d** Hi-C resolution of the inactive chromosome $X^{mus}$ achieved in this study, calculated as described previously[2]. Resolution of comparable studies assessing the inactive X using allele-specific *in situ* Hi-C are shown[3,4]. **e** Mega-domain strength on the active $X^{cas}$ depicted by the PC1 of the Hi-C matrices without splitting at *Dxz4*. Lines show smoothed data from a fitted loess curve with span 0.25. Shading denotes 95% confidence interval. Dotted line indicates position of *Dxz4.* **f** A/B compartments of chromosome X at 100-kb resolution obtained with principal component analysis of matrices split at the *Dxz4* mega-domain boundary of neural precursor cells (NPCs)[3] and mouse embryonic fibroblasts (MEFs)[4]. Positive PC1 values represent A-like compartments (red); negative PC1 values represent B-like compartments (blue).

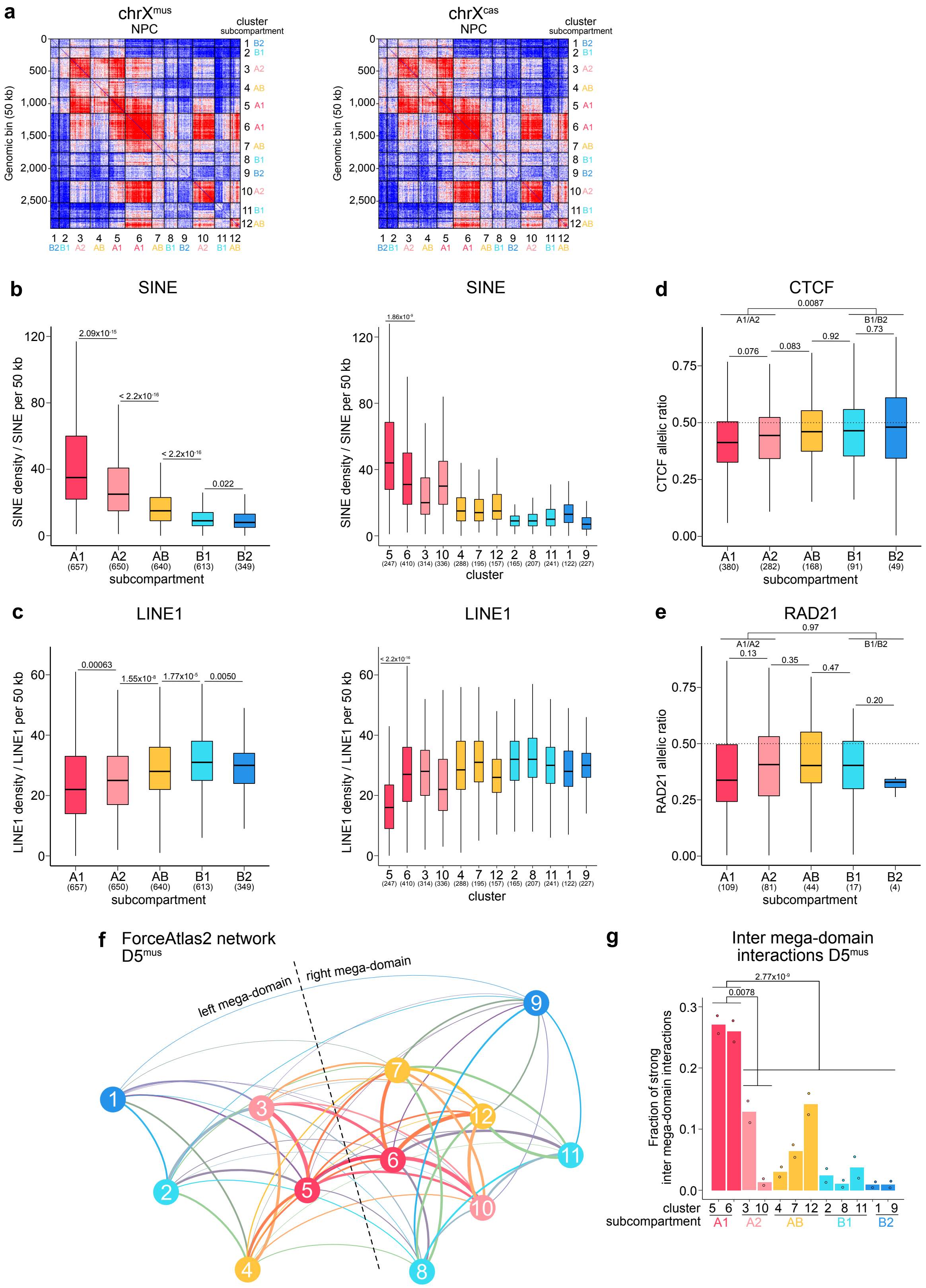


**Supplementary Fig. 3 Subcompartmentalization of the inactive X chromosome.**
(legend on next page)

**Supplementary Fig. 3 Subcompartmentalization of the inactive X chromosome. a** Interaction matrices of spatial clusters at 50-kb resolution. **b** Density of SINE repeats per 50 kb bin for subcompartments and spatial clusters. **c** Density of LINE1 repeats per 50 kb bin for subcompartments and spatial clusters. **d** Allelic ratio of CTCF peaks between $NPC^{mus}$ and $NPC^{cas}$ of subcompartments. ChIP-seq data from[3]. **e** Allelic ratio of RAD21 peaks between $NPC^{mus}$ and $NPC^{cas}$ of subcompartments. ChIP-seq data from[3]. (**b**-**e**) The numbers above the bars indicate p-values (two-sample unpaired Wilcoxon-Mann-Whitney test with R defaults). Box plots depict the first and third quartiles as the lower and upper bounds of the box, with a band inside the box showing the median value and whiskers representing 1.5x the interquartile range. n is given in brackets and indicates number of 50 kb bins. **f** Network of spatial clusters on chromosome $X^{mus}$ in D5 P-RFP+ obtained by applying the ForceAtlas2 algorithm to Hi-C interaction patterns of spatial clusters. Each cluster represents a single node of the network. Line-width correlates with interaction strength. **g** Inter-mega-domain interactions of clusters (across the mega-domain boundary) in D5 P-RFP+$^{mus}$. The numbers above the bars indicate p-values (unpaired two-sided t-test with R defaults). n = 2 biologically independent replicates.

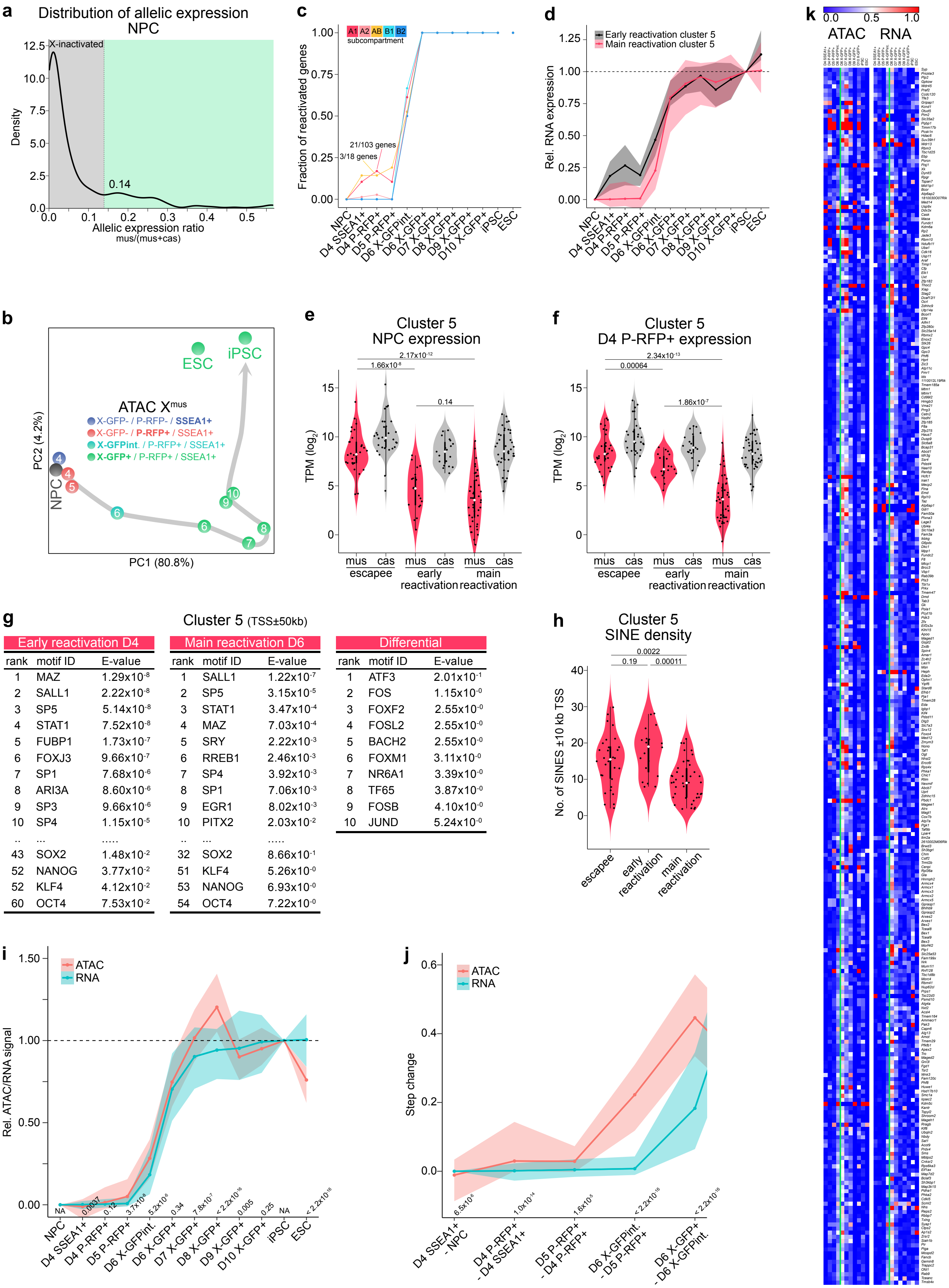


g

Cluster 5 (TSS±50kb)

| Early reactivation D4 | | |
|---|---|---|
| rank | motif ID | E-value |
| 1 | MAZ | $1.29\times10^{-8}$ |
| 2 | SALL1 | $2.22\times10^{-8}$ |
| 3 | SP5 | $5.14\times10^{-8}$ |
| 4 | STAT1 | $7.52\times10^{-8}$ |
| 5 | FUBP1 | $1.73\times10^{-7}$ |
| 6 | FOXJ3 | $9.66\times10^{-7}$ |
| 7 | SP1 | $7.68\times10^{-6}$ |
| 8 | ARI3A | $8.60\times10^{-6}$ |
| 9 | SP3 | $9.66\times10^{-6}$ |
| 10 | SP4 | $1.15\times10^{-5}$ |
| .. | ... | ..... |
| 43 | SOX2 | $1.48\times10^{-2}$ |
| 52 | NANOG | $3.77\times10^{-2}$ |
| 52 | KLF4 | $4.12\times10^{-2}$ |
| 60 | OCT4 | $7.53\times10^{-2}$ |

| Main reactivation D6 | | |
|---|---|---|
| rank | motif ID | E-value |
| 1 | SALL1 | $1.22\times10^{-7}$ |
| 2 | SP5 | $3.15\times10^{-5}$ |
| 3 | STAT1 | $3.47\times10^{-4}$ |
| 4 | MAZ | $7.03\times10^{-4}$ |
| 5 | SRY | $2.22\times10^{-3}$ |
| 6 | RREB1 | $2.46\times10^{-3}$ |
| 7 | SP4 | $3.92\times10^{-3}$ |
| 8 | SP1 | $7.06\times10^{-3}$ |
| 9 | EGR1 | $8.02\times10^{-3}$ |
| 10 | PITX2 | $2.03\times10^{-2}$ |
| .. | ... | ..... |
| 32 | SOX2 | $8.66\times10^{-1}$ |
| 51 | KLF4 | $5.26\times10^{-0}$ |
| 53 | NANOG | $6.93\times10^{-0}$ |
| 54 | OCT4 | $7.22\times10^{-0}$ |

| Differential | | |
|---|---|---|
| rank | motif ID | E-value |
| 1 | ATF3 | $2.01\times10^{-1}$ |
| 2 | FOS | $1.15\times10^{-0}$ |
| 3 | FOXF2 | $2.55\times10^{-0}$ |
| 4 | FOSL2 | $2.55\times10^{-0}$ |
| 5 | BACH2 | $2.55\times10^{-0}$ |
| 6 | FOXM1 | $3.11\times10^{-0}$ |
| 7 | NR6A1 | $3.39\times10^{-0}$ |
| 8 | TF65 | $3.87\times10^{-0}$ |
| 9 | FOSB | $4.10\times10^{-0}$ |
| 10 | JUND | $5.24\times10^{-0}$ |

**Supplementary Fig. 4 Initiation of chromatin opening and gene expression from a distinct 3D cluster.**

(legend on next page)

**Supplementary Fig. 4 Initiation of chromatin opening and gene expression from a distinct 3D cluster. a** Distribution of the allelic expression ratio of chromosome X in NPCs (n = 275). The dashed line represents the cut-off of 0.14, above which genes were considered biallelically expressed (green shading). Only protein-coding genes with sufficient allelic information, expression for chromosome $X^{cas}$, and biallelic expression in iPSC and ESC are counted (see methods). **b** PCA of dynamics of chromatin opening on the $X^{mus}$ during reprogramming (n = 570,104 50 kb bins). Grey arrow, hypothetical trajectory. **c** Dynamics of gene reactivation of subcompartments. Fractions of reactivated genes per cluster are shown. 0, no reactivated gene. 1, all genes reactivated. Threshold for gene reactivation, allelic expression ratio >0.14, see (**a**). While subcompartment AB shows a similar fraction of early reactivating genes to A1, it is biased by the low number of genes in this subcompartment, with a total of 18 genes and 3 reactivating at D4 P-RFP+. **d** Relative RNA expression (NPC = 0, iPSC = 1) for genes of cluster 5 reactivating early, at D4 P-RFP+, compared to genes reactivating after that ("main reactivation"). Line shows median. Shading denotes 50% confidence interval. **e** Violin plots showing the RNA expression from the mus allele of genes of cluster 5 in NPCs. TPM, transcripts per million. The numbers above the bars indicate p-values (two-sample unpaired Wilcoxon-Mann-Whitney test with R defaults). **f** As (**e**) in D4 P-RFP+ cells. **g** Enrichment of transcription factor motifs. AME was used to identify differentially enriched motifs from ATAC-seq peaks in a window of +- 50 kb around the TSS of cluster 5 genes reactivating early or after that. Differential AME analysis for early reactivation-specific motifs was performed using peaks around early reactivating genes as primary sequence and peaks around main reactivating genes as control. **h** Violin plots showing the number of SINE repeats in a window of +-10 kb around the TSS of genes of cluster 5. The numbers above the bars indicate p-values (two-sample unpaired Wilcoxon-Mann-Whitney test with R defaults). **i** Relative gene expression and chromatin opening at gene promoters compared (NPC = 0, iPSC = 1). Line shows median. Shading denotes 50% confidence interval. The numbers above the axis indicate p-values (two-sample unpaired Wilcoxon-Mann-Whitney test with R defaults). n = 293. **j** Step changes of gene expression and chromatin opening at gene promoters compared. Line shows median. Shading denotes 50% confidence interval. The numbers above the axis indicate p-values (two-sample unpaired Wilcoxon-Mann-Whitney test with R defaults). n = 293. **k** Step changes of gene expression and chromatin opening at gene promoters compared. Genes are sorted by their chromosomal position. Vertical green line indicates main X-reactivation in the D6 X-GFP+ population. The color gradient represents the step change. Blue, 0. White, 0.5. Red, 1. n = 293.

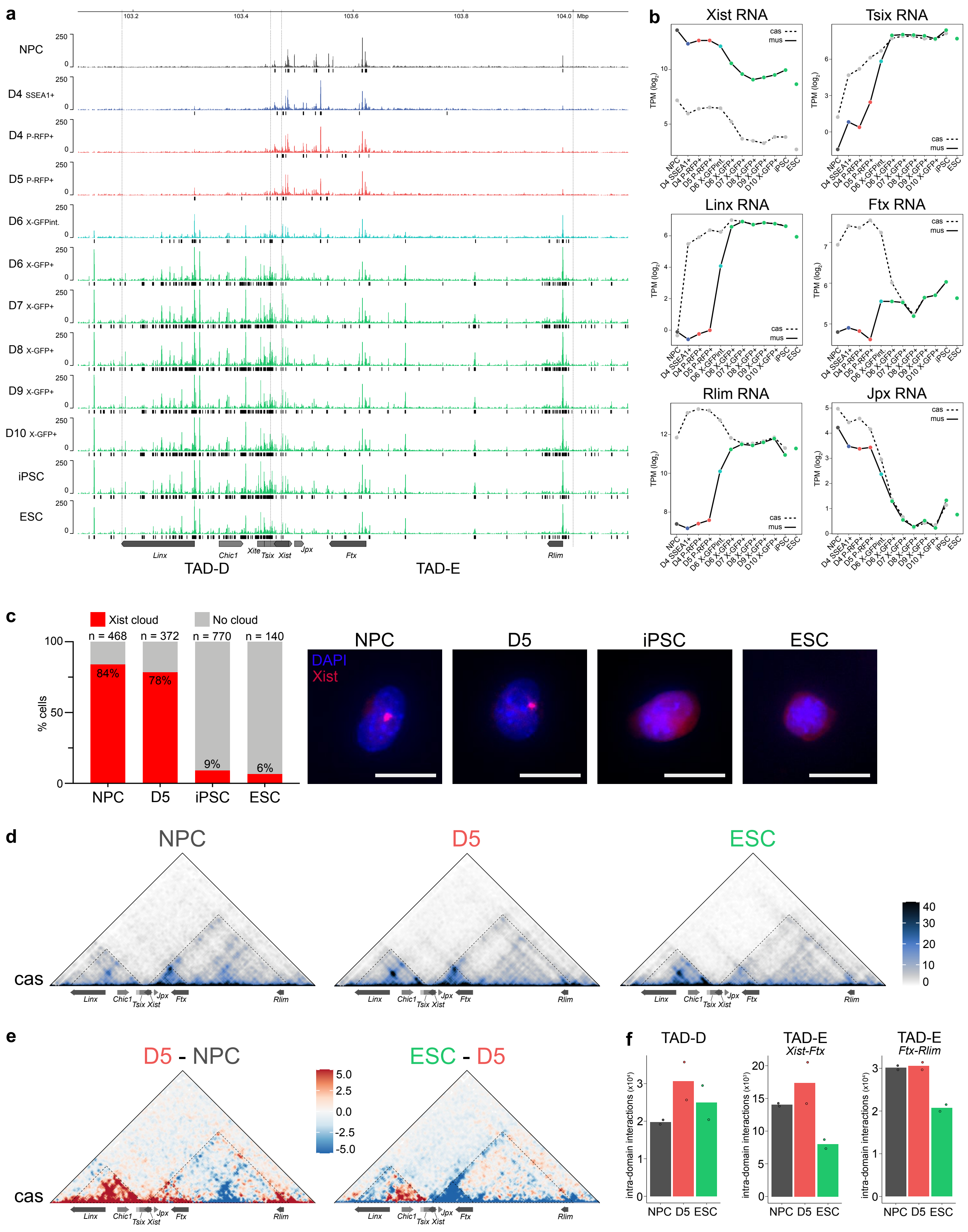


**Supplementary Fig. 5 Remodelling of the X-inactivation centre leading to *Xist* downregulation.**

(legend on next page)

**Supplementary Fig. 5 Remodelling of the X-inactivation centre leading to *Xist* downregulation.** **a** ATAC-seq profiles at a region encompassing *Tsix* TAD-D (mm10; 103.18 Mb - 103.45 Mb) and *Xist* TAD-E (mm10; 103.47 Mb - 104.0 Mb). ATAC-peaks in black (except for NPCs, differential peaks compared to NPCs are shown). Only genes with implicated roles in X-inactivation or X-reactivation are shown. **b** RNA expression of *Xist*, *Tsix*, *Linx*, *Ftx*, *Rlim* and *Jpx* ($X^{mus}$ and $X^{cas}$).
**c** Downregulation of Xist RNA during reprogramming based on RNA-FISH. Percentages of cells containing Xist RNA FISH clouds are shown for one of two independent experiments. Scale bar, 5 µm. **d** Allele-specific Hi-C maps of chromosome $X^{cas}$ at 10-kb resolution at a region encompassing TAD-D (left) and TAD-E (right). **e** Differential allele-specific Hi-C maps of chromosome $X^{cas}$ at 10-kb resolution at a region encompassing TAD-D and TAD-E. (**d, e**) Dotted lines show TAD borders and additionally separate TAD-E in two regions at the TSS of *Ftx* (mm10; 103.62 Mb) for quantification in (**f**). **f** Sum of intra-domain interactions of $X^{cas}$ are shown. TAD-E was separated in two regions at the TSS of *Ftx*. n = 2 biologically independent replicates.

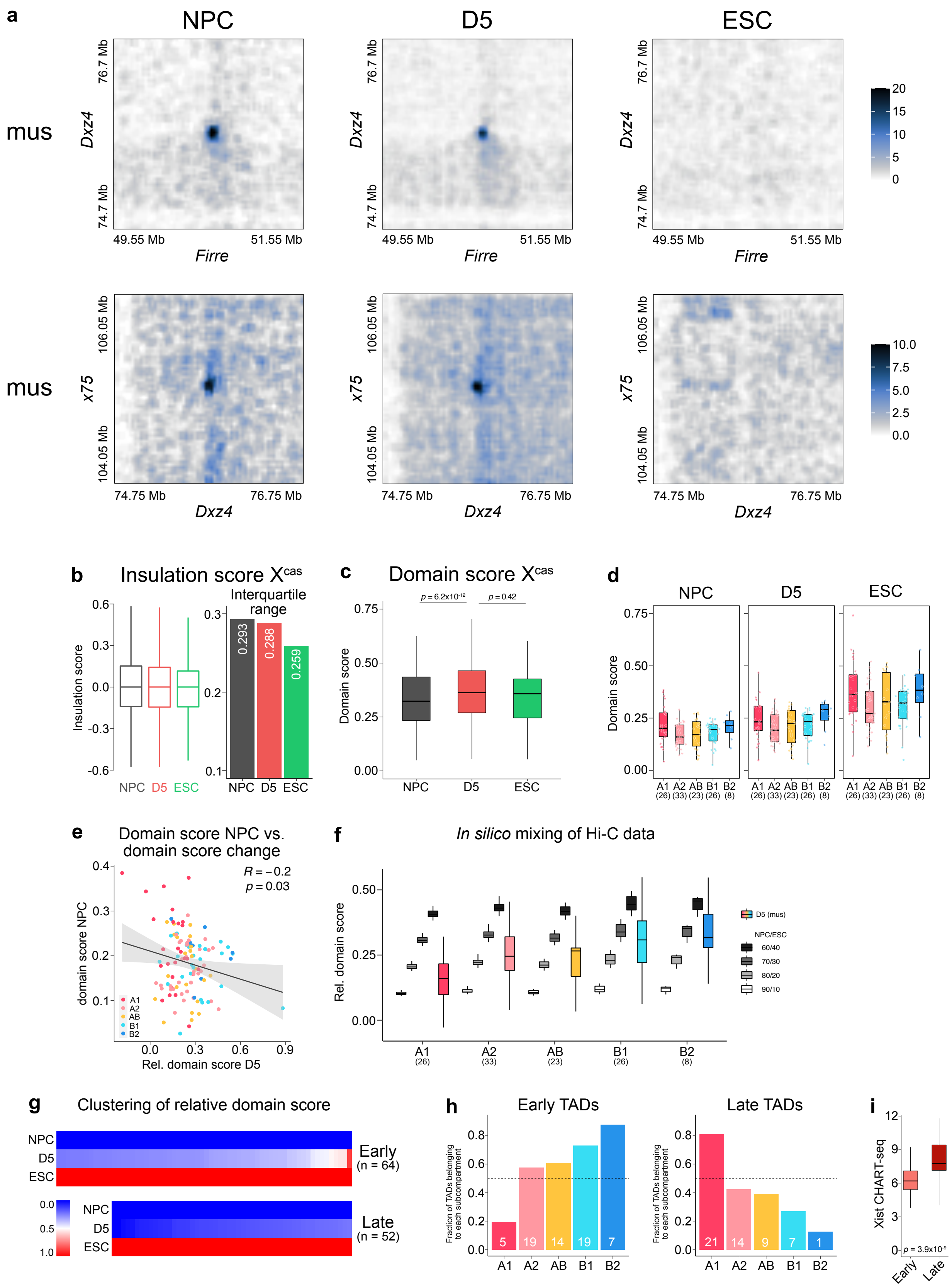


**Supplementary Fig. 6 Structural changes during X-reactivation in the absence of chromatin opening and transcription.**
(legend on next page)

**Supplementary Fig. 6 Structural changes during X-reactivation in the absence of chromatin opening and transcription. a** Allele-specific Hi-C maps at 30-kb resolution showing superloop interactions between *Dxz4* and *Firre*, as well as *x75* and *Dxz4* ± 1 Mb. **b** Comparison of insulation scores for chromosome $X^{cas}$. Interquartile range of insulation scores is shown on the right. n = 2,785 50 kb bins. **c** Comparison of domain scores for chromosome $X^{cas}$. The numbers above the bars indicate p-values (two-sample unpaired Wilcoxon-Mann-Whitney test with R defaults). n = 100 TADs. **d** Domain scores for $X^{mus}$ of subcompartments. n is given in brackets and indicates number of TADs. **e** Correlation between the absolute domain score in NPCs and the relative domain score at D5 is shown. Points represent TADs. Colours of points indicate subcompartments. R and p-values calculated by Pearson's correlation are shown. Black line represents linear regression fitting. Shading denotes 95% confidence interval of the fit. **f** relative domain score is shown for *in silico* mixed Hi-C matrices of $NPC^{mus}$ with $ESC^{mus}$ at given ratios and compared to the relative domain score of $D5^{mus}$. n is given in brackets and indicates number of TADs. **g** *k*-means clustering (*k* = 2) of relative domain scores to identify early and late TADs. **h** Comparison of the fraction of TADs of each subcompartment belonging to early or late TADs. Absolute numbers of TADs for each subcompartment are given in the barplots. **i** Xist RNA enrichment of early and late TADs in NPCs (composite scaled data). CHART-seq data from[3]. The numbers above the bars indicate p-values (two-sample unpaired Wilcoxon-Mann-Whitney test with R defaults). n = 64 early TAD and 52 late TADs. (**b**,**c**,**d**,**f**,**i**) Box plots depict the first and third quartiles as the lower and upper bounds of the box, with a band inside the box showing the median value and whiskers representing 1.5x the interquartile range.

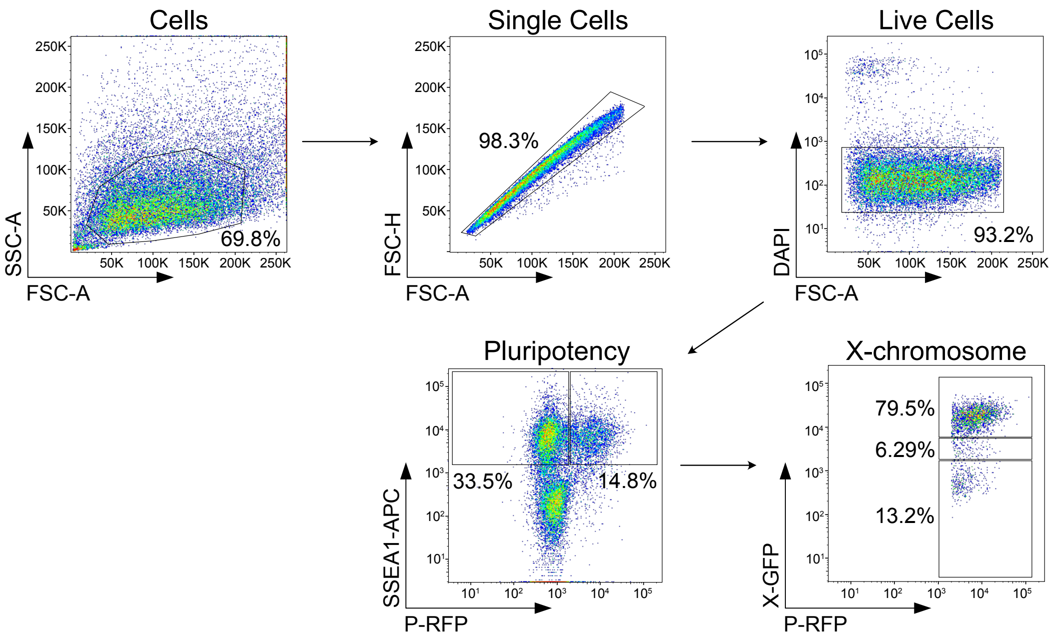


**Supplementary Fig. 7 FACS gating strategy.** The gating was performed as follows, exemplified for day 8 iPSCs: First, forward and side scatter were used to gate out debris. Second, forward scatter height and area were used to gate out doublets. Third, DAPI-positivity was used to gate out dead cells. Fourth, pluripotent cells were then defined on live cells by SSEA1-APC+ or SSEA1-APC+/P-RFP+. Last, on pluripotent cells, cells corresponding to different X-status were defined using X-GFP. Boundaries between pluripotent and non-pluripotent populations, as well as between X-reactivated and non-reactivated populations were defined using non stained cells and cell lines without fluorescent reporters as negative controls.

**Supplementary Table 1**. Number of normalized allelic reads of each *in situ* Hi-C dataset generated in this study.

| sample | replicate | chr X (mus) | chr X (cas) | chr 13 (mus) | chr 13 (cas) |
|---|---|---|---|---|---|
| NPC | 1 | 6,087,754 | 7,498,096 | 10,774,993 | 10,618,036 |
| NPC | 2 | 3,043,877 | 3,749,048 | 14,484,897 | 14,364,026 |
| D5 P-RFP+ | 1 | 12,143,539 | 9,244,472 | 13,843,684 | 13,408,834 |
| D5 P-RFP+ | 2 | 6,071,770 | 4,622,236 | 6,921,842 | 20,098,602 |
| ESC | 1 | 10,432,393 | 10,163,224 | 10,774,993 | 10,618,036 |
| ESC | 2 | 5,216,196 | 5,081,612 | 14,484,897 | 14,364,026 |

**Supplementary Table 2** List of primers used in this study.

| Sequence | Target gene/region | Orientation | Application |
|---|---|---|---|
| ATGAATACGGCTACAGCAACAGG | *Gapdh* | Forward | quantitative RT-PCR |
| CTCTTGCTCAGTGTCCTTGCTG | *Gapdh* | Reverse | quantitative RT-PCR |
| AGGAAACGACGAGAACAGTTGA | MKOS cMyc-Klf4 | Forward | quantitative RT-PCR |
| GACGCAGTGTCTTCTCCCTTC | MKOS cMyc-Klf4 | Reverse | quantitative RT-PCR |
| GGACCACCTTGCCTTACACAT | MKOS Klf4-Oct4 | Forward | quantitative RT-PCR |
| GAAGCTTAGCCAGGTTCGAGA | MKOS Klf4-Oct4 | Reverse | quantitative RT-PCR |
| ACCACACTCTACTCAGTCCCT | MKOS Oct4-Sox2 | Forward | quantitative RT-PCR |
| AGCTCCGTCTCCATCATGTT | MKOS Oct4-Sox2 | Reverse | quantitative RT-PCR |
| CTACCACCGATTCTATGCCCC | rtTA | Forward | quantitative RT-PCR |
| CGCTTTCGCACTTTAGCTGTT | rtTA | Reverse | quantitative RT-PCR |
| TTAGCCAGGCAGCTAGAGGA | *Jpx* | Forward | quantitative RT-PCR |
| AGCCGTATTCCTCCATGGTT | *Jpx* | Reverse | quantitative RT-PCR |
| ATCATACTAAAGGCCACACAAAGAAT | *Xist* (mus) | Forward | quantitative RT-PCR |
| ATTTGGATTGCAAGGTGGAT | *Xist* (mus) | Reverse | quantitative RT-PCR |

# Supplementary References


1. Yang, T. *et al.* HiCRep: assessing the reproducibility of Hi-C data using a stratum-adjusted correlation coefficient. *Genome Res.* **27**, 1939–1949 (2017).
2. Rao, S. S. P. *et al.* A 3D map of the human genome at kilobase resolution reveals principles of chromatin looping. *Cell* **159**, 1665–1680 (2014).
3. Wang, C.-Y., Jégu, T., Chu, H.-P., Oh, H. J. & Lee, J. T. SMCHD1 Merges Chromosome Compartments and Assists Formation of Super-Structures on the Inactive X. *Cell* vol. 174 406–421.e25 (2018).
4. Wang, C.-Y., Colognori, D., Sunwoo, H., Wang, D. & Lee, J. T. PRC1 collaborates with SMCHD1 to fold the X-chromosome and spread Xist RNA between chromosome compartments. *Nat. Commun.* **10**, 2950 (2019).
