# Supplementary material for: Chromosome compartments on the inactive X guide TAD formation independently of transcription during X-reactivation
Source: Nat Commun. 2021 Jun 9;12:3499. doi: 10.1038/s41467-021-23610-1 (PMC8190187; doi:10.1038/s41467-021-23610-1)
Supplement: Supplementary file 3 — Reporting Summary [file 41467_2021_23610_MOESM3_ESM.pdf]

nature research

Corresponding author(s): Bernhard Payer, Guillaume Filion

Last updated by author(s): Apr 9, 2021

Reporting Summary

Nature Research wishes to improve the reproducibility of the work that we publish. This form provides structure for consistency and transparency in reporting. For further information on Nature Research policies, see our Editorial Policies and the Editorial Policy Checklist.

Statistics

For all statistical analyses, confirm that the following items are present in the figure legend, table legend, main text, or Methods section.

n/a	Confirmed	
☐	☒	The exact sample size (n) for each experimental group/condition, given as a discrete number and unit of measurement
☐	☒	A statement on whether measurements were taken from distinct samples or whether the same sample was measured repeatedly
☐	☒	The statistical test(s) used AND whether they are one- or two-sided<br>*Only common tests should be described solely by name; describe more complex techniques in the Methods section.*
☒	☐	A description of all covariates tested
☐	☒	A description of any assumptions or corrections, such as tests of normality and adjustment for multiple comparisons
☐	☒	A full description of the statistical parameters including central tendency (e.g. means) or other basic estimates (e.g. regression coefficient) AND variation (e.g. standard deviation) or associated estimates of uncertainty (e.g. confidence intervals)
☐	☒	For null hypothesis testing, the test statistic (e.g. F, t, r) with confidence intervals, effect sizes, degrees of freedom and P value noted<br>*Give P values as exact values whenever suitable.*
☒	☐	For Bayesian analysis, information on the choice of priors and Markov chain Monte Carlo settings
☐	☒	For hierarchical and complex designs, identification of the appropriate level for tests and full reporting of outcomes
☐	☒	Estimates of effect sizes (e.g. Cohen's d, Pearson's r), indicating how they were calculated

Our web collection on statistics for biologists contains articles on many of the points above.

Software and code

Policy information about availability of computer code

Data collection	No softwares were used to collect the data
Data analysis	asmap (https://github.com/gui11aume/asmap/tree/v1.0.0), BEDTools (2.29.0, Quinlan and Hall, 2010), CrossMap (0.3.8, Zhao et al., 2014), DESeq2 (1.28.1, Love et al., 2014), FastQC (http://www.bioinformatics.babraham.ac.uk/projects/fastqc), Fiji (2.1.0, Schindelin et al., 2012), FlowJo (version 10.7.1 for macOS), mclust (5.4.7, Scrucca et al., 2016), OneD (Vidal et al. 2018, included in R package dryhic), SAMtools (Li et al., 2009), STAR (Dobin et al., 2013), sva R package (3.22), TADbit (Serra et al., 2017), Trimmomatic (Bolger et al., 2014), wig2bed (Neph et al., 2012), tidyverse (1.3.0, Wickham et al., 2019), t.test() R function, wilcox.test() R function, Zerone (Cuscó and Filion, 2016)

For manuscripts utilizing custom algorithms or software that are central to the research but not yet described in published literature, software must be made available to editors and reviewers. We strongly encourage code deposition in a community repository (e.g. GitHub). See the Nature Research guidelines for submitting code & software for further information.

Data

Policy information about availability of data

All manuscripts must include a data availability statement. This statement should provide the following information, where applicable:

- Accession codes, unique identifiers, or web links for publicly available datasets
- A list of figures that have associated raw data
- A description of any restrictions on data availability

The sequencing data sets generated during the current study are deposited in the Gene Expression Omnibus (GEO) GSE157448. ChIP-seq and Xist CHART-seq datasets used in the current study are available in the GEO database under accession number GSE99991.

Field-specific reporting

Please select the one below that is the best fit for your research. If you are not sure, read the appropriate sections before making your selection.

- [x] Life sciences
- [ ] Behavioural & social sciences
- [ ] Ecological, evolutionary & environmental sciences

For a reference copy of the document with all sections, see nature.com/documents/nr-reporting-summary-flat.pdf

Life sciences study design

All studies must disclose on these points even when the disclosure is negative.

Sample size	Sample size was not determined by calculations. Sequencing experiments were carried out with two biological replicates with two technical replicates each to avoid technical issues influencing data interpretation.
Data exclusions	TAD border detection criteria were pre-established in order to increase the accuracy of the TAD borders detection. All read and bin filtering strategies used for Hi-C data analysis are described in detail in the Method section.
Replication	All sequencing experiments were performed in n=2 biologically independent experiments with n=2 technical replicates per biological replicate. No replicates were excluded from analyses presented, and all attempts at replication were successful. LNA knockdown experiments were performed in n = 4 biologically independent experiments. All attempts at replication were successful for those experiments.
Randomization	Randomization is not relevant to this study because no comparisons between experimental groups were made.
Blinding	Blinding was not relevant to this study because all metrics were derived from absolute quantitative methods without human subjectivity.

Reporting for specific materials, systems and methods

We require information from authors about some types of materials, experimental systems and methods used in many studies. Here, indicate whether each material, system or method listed is relevant to your study. If you are not sure if a list item applies to your research, read the appropriate section before selecting a response.

Materials & experimental systems

n/a	Involved in the study	
[ ]	[x]	Antibodies
[ ]	[x]	Eukaryotic cell lines
[x]	[ ]	Palaeontology and archaeology
[x]	[ ]	Animals and other organisms
[x]	[ ]	Human research participants
[x]	[ ]	Clinical data
[x]	[ ]	Dual use research of concern

Methods

n/a	Involved in the study	
[ ]	[x]	ChIP-seq
[ ]	[x]	Flow cytometry
[x]	[ ]	MRI-based neuroimaging

Antibodies

Antibodies used	SSEA1 Monoclonal Antibody (eBioMC-480 (MC-480)), eFluor 660, eBioscience™, # 50-8813-41, Invitrogen
Validation	This eBioMC-480 (MC-480) antibody has been pre-titrated and tested by intracellular staining and flow cytometric analysis of the F9 cell line. The antibody has been previously used to detect and isolate mouse ESC using FACS (ten Berge et al. 2011; Fragola et al. 2013; Das et al. 2014; Zhang et al. 2014; Cirera-Salinas et al. 2017)

Eukaryotic cell lines

Policy information about cell lines

Cell line source(s)	We used the previously established ESC line EL16.7 TST (Ogawa et al., 2008). The EL16.7 TST cell line has been obtained from Jeannie Lee, Massachusetts General Hospital (Boston, USA).
Authentication	The cell line was validated, and the presence of two X chromosomes confirmed, by karyotyping and DNA FISH probes against the X chromosome and by comparing the allelic transcriptome with previous published data.
Mycoplasma contamination	Cells were monthly tested for mycoplasma contamination using PCR. Cells always tested negative.
Commonly misidentified lines (See ICLAC register)	No commonly misidentified cell lines were used.

ChIP-seq

Data deposition

- [x] Confirm that both raw and final processed data have been deposited in a public database such as GEO.
- [x] Confirm that you have deposited or provided access to graph files (e.g. BED files) for the called peaks.

Data access links<br>*May remain private before publication.*	https://www.ncbi.nlm.nih.gov/geo/query/acc.cgi?acc=GSE157448
Files in database submission	ATAC-seq – NPC rep 1<br>ATAC-seq – NPC rep 2<br>ATAC-seq – NPC rep 3<br>ATAC-seq – NPC rep 4<br>ATAC-seq – D4 SSEA1+ rep 1<br>ATAC-seq – D4 SSEA1+ rep 2<br>ATAC-seq – D4 SSEA1+ rep 3<br>ATAC-seq – D4 SSEA1+ rep 4<br>ATAC-seq – D4 P-RFP+ rep 1<br>ATAC-seq – D4 P-RFP+ rep 2<br>ATAC-seq – D4 P-RFP+ rep 3<br>ATAC-seq – D4 P-RFP+ rep 4<br>ATAC-seq – D5 P-RFP+ rep 1<br>ATAC-seq – D5 P-RFP+ rep 2<br>ATAC-seq – D5 P-RFP+ rep 3<br>ATAC-seq – D5 P-RFP+ rep 4<br>ATAC-seq – D6 P-RFP+ rep 1<br>ATAC-seq – D6 P-RFP+ rep 2<br>ATAC-seq – D6 P-RFP+ rep 3<br>ATAC-seq – D6 P-RFP+ rep 4<br>ATAC-seq – D6 X-GFPint rep 1<br>ATAC-seq – D6 X-GFPint rep 2<br>ATAC-seq – D6 X-GFPint rep 3<br>ATAC-seq – D6 X-GFPint rep 4<br>ATAC-seq – D6 X-GFP+ rep 1<br>ATAC-seq – D6 X-GFP+ rep 2<br>ATAC-seq – D6 X-GFP+ rep 3<br>ATAC-seq – D6 X-GFP+ rep 4<br>ATAC-seq – D7 X-GFP+ rep 1<br>ATAC-seq – D7 X-GFP+ rep 2<br>ATAC-seq – D7 X-GFP+ rep 3<br>ATAC-seq – D7 X-GFP+ rep 4<br>ATAC-seq – D8 X-GFP+ rep 1<br>ATAC-seq – D8 X-GFP+ rep 2<br>ATAC-seq – D8 X-GFP+ rep 3<br>ATAC-seq – D8 X-GFP+ rep 4<br>ATAC-seq – D9 X-GFP+ rep 1<br>ATAC-seq – D9 X-GFP+ rep 2<br>ATAC-seq – D9 X-GFP+ rep 3<br>ATAC-seq – D9 X-GFP+ rep 4<br>ATAC-seq – D10 X-GFP+ rep 1<br>ATAC-seq – D10 X-GFP+ rep 2<br>ATAC-seq – D10 X-GFP+ rep 3<br>ATAC-seq – D10 X-GFP+ rep 4<br>ATAC-seq – iPSC rep 1<br>ATAC-seq – iPSC rep 2<br>ATAC-seq – iPSC rep 3<br>ATAC-seq – iPSC rep 4<br>ATAC-seq – ESC rep 1<br>ATAC-seq – ESC rep 2<br>ATAC-seq – ESC rep 3<br>ATAC-seq – ESC rep 4<br>RNA-seq – NPC rep 1<br>RNA-seq – NPC rep 2<br>RNA-seq – NPC rep 3<br>RNA-seq – NPC rep 4<br>RNA-seq – D4 SSEA1+ rep 1

RNA-seq – D4 SSEA1+ rep 2
RNA-seq – D4 SSEA1+ rep 3
RNA-seq – D4 SSEA1+ rep 4
RNA-seq – D4 P-RFP+ rep 1
RNA-seq – D4 P-RFP+ rep 2
RNA-seq – D4 P-RFP+ rep 3
RNA-seq – D4 P-RFP+ rep 4
RNA-seq – D5 P-RFP+ rep 1
RNA-seq – D5 P-RFP+ rep 2
RNA-seq – D5 P-RFP+ rep 3
RNA-seq – D5 P-RFP+ rep 4
RNA-seq – D6 P-RFP+ rep 1
RNA-seq – D6 P-RFP+ rep 2
RNA-seq – D6 P-RFP+ rep 3
RNA-seq – D6 P-RFP+ rep 4
RNA-seq – D6 X-GFPint rep 1
RNA-seq – D6 X-GFPint rep 2
RNA-seq – D6 X-GFPint rep 3
RNA-seq – D6 X-GFPint rep 4
RNA-seq – D6 X-GFP+ rep 1
RNA-seq – D6 X-GFP+ rep 2
RNA-seq – D6 X-GFP+ rep 3
RNA-seq – D6 X-GFP+ rep 4
RNA-seq – D7 X-GFP+ rep 1
RNA-seq – D7 X-GFP+ rep 2
RNA-seq – D7 X-GFP+ rep 3
RNA-seq – D7 X-GFP+ rep 4
RNA-seq – D8 X-GFP+ rep 1
RNA-seq – D8 X-GFP+ rep 2
RNA-seq – D8 X-GFP+ rep 3
RNA-seq – D8 X-GFP+ rep 4
RNA-seq – D9 X-GFP+ rep 1
RNA-seq – D9 X-GFP+ rep 2
RNA-seq – D9 X-GFP+ rep 3
RNA-seq – D9 X-GFP+ rep 4
RNA-seq – D10 X-GFP+ rep 1
RNA-seq – D10 X-GFP+ rep 2
RNA-seq – D10 X-GFP+ rep 3
RNA-seq – D10 X-GFP+ rep 4
RNA-seq – iPSC rep 1
RNA-seq – iPSC rep 2
RNA-seq – iPSC rep 3
RNA-seq – iPSC rep 4
RNA-seq – ESC rep 1
RNA-seq – ESC rep 2
RNA-seq – ESC rep 3
RNA-seq – ESC rep 4
Hi-C – NPC rep 1
Hi-C – NPC rep 2
Hi-C – NPC rep 3
Hi-C – NPC rep 4
Hi-C – D5 P-RFP+ rep 1
Hi-C – D5 P-RFP+ rep 2
Hi-C – D5 P-RFP+ rep 3
Hi-C – D5 P-RFP+ rep 4
Hi-C – ESC rep 1
Hi-C – ESC rep 2
Hi-C – ESC rep 3
Hi-C – ESC rep 4

Genome browser session
(e.g. UCSC)

Provide a link to an anonymized genome browser session for "Initial submission" and "Revised version" documents only, to enable peer review. Write "no longer applicable" for "Final submission" documents.

Methodology

Replicates

Two biological replicates, detailed information in Methods section

Sequencing depth

On average, approximately 30-40 million paired-end 125 bp reads were generated per every ATAC-seq sample. On average, approximately 30-40 million paired-end 125 bp reads were generated per every RNA-seq sample. On average, approximately

	500-1,000 million paired-end 125 bp reads were generated per every Hi-C sample.
Antibodies	SSEA1 Monoclonal Antibody (eBioMC-480 (MC-480)), eFluor 660, eBioscience™, # 50-8813-41, Invitrogen
Peak calling parameters	Zerone/make atac/confidence score > 99.9%
Data quality	FastQC
Software	Zerone (Cuscó and Filion, 2016)

Flow Cytometry

Plots

Confirm that:

☒ The axis labels state the marker and fluorochrome used (e.g. CD4-FITC).

☒ The axis scales are clearly visible. Include numbers along axes only for bottom left plot of group (a 'group' is an analysis of identical markers).

☒ All plots are contour plots with outliers or pseudocolor plots.

☒ A numerical value for number of cells or percentage (with statistics) is provided.

Methodology

Sample preparation	Cells were dissociated using Accutase (Thermo Fisher Scientific, 00-4555-56) (for NPCs), 0.05% Trypsin-EDTA (Thermo Fisher Scientific, 25300054) (for ESCs) or 0.25% Trypsin-EDTA (Thermo Fisher Scientific, 25200056) (for iPSCs) and then stained with SSEA-1 eFluor 660 (Thermo Fisher Scientific, 50-8813-42) for 15 min at 4°C. Cells were washed once with 0.5% BSA in PBS and then FACS sorted.
Instrument	FACS sorting was performed using either a BD FACSAria II SORP or a BD Influx. FACS analysis was performed using a BD LSRFortessa.
Software	Data analysis was performed using FlowJo v10.
Cell population abundance	ESC were routinely FACS sorted for P-RFP+/X-GFP+ and showed >95% double reporter expression post-sort. No post-sort fractions were collected for NPC and reprogramming experiments to omit loss of samples.
Gating strategy	Gating strategy is exemplified in Supplementary Fig. 7. First, forward and side scatter were used to gate out debris. Second, forward scatter height and area were used to gate out doublets. Third, DAPI-positivity was used to gate out dead cells. Fourth, pluripotent cells were then defined on live cells by SSEA1-APC+ or SSEA1-APC+/P-RFP+. Last, on pluripotent cells, cells corresponding to different X-status were defined using X-GFP. Boundaries between pluripotent and non-pluripotent populations, as well as between X-reactivated and non-reactivated populations were defined using non stained cells and cell lines without fluorescent reporters as negative controls.

☒ Tick this box to confirm that a figure exemplifying the gating strategy is provided in the Supplementary Information.
